# The impact of additional cytogenetic abnormalities at diagnosis and during therapy with tyrosine kinase inhibitors in Chronic Myeloid Leukaemia

**Published:** 2015

**Authors:** AM Crisan, D Coriu, C Arion, A Colita, C Jardan

**Affiliations:** * “Carol Davila” University of Medicine and Pharmacy, Bucharest, Romania; ** Fundeni Clinical Institute, Bucharest, Romania

**Keywords:** CML, TKI, additional cytogenetic abnormalities, Philadelphia chromosome

## Abstract

**Background:** Chronic Myeloid Leukemia’s (CML) treatment was optimized since the development of tyrosine kinase inhibitors (TKI) and an increased overall survival during TKI was noticed. During the TKI era, protocols for assessing response and resistance to treatment were developed. Additional chromosomal abnormalities (ACAs) are strongly associated with disease progression but their prognostic impact and influence on treatment response are yet to be defined. The aim of this study was to analyze the impact of ACAs on time to achieve complete cytogenetic response (CCyR), treatment and overall survival.

**Materials and methods:** Since 2005 until 2013, the data from the Hematology and Bone Marrow Transplantation Department of Fundeni Clinical Institute was collected. In this observational retrospective single centre study, 28 CML patients with ACAs at diagnosis and during TKI treatment were included.

**Results:** From ACAs at diagnosis group, the most frequent major route ACAs were trisomy 8, trisomy 19 and second Philadelphia (Ph) chromosome and the most frequent minor route ACAs were monosomies and structural abnormalities (inversions and translocations). From the ACAs during the TKI group, the most frequent major route cytogenetic abnormalities in Ph positive and negative cells were trisomy 8, trisomy 19 and second Ph chromosome and the most frequent minor route cytogenetic abnormalities in Ph positive and negative cells were marker chromosomes and structural abnormalities (inversions, translocations and dicentric chromosomes).

**Conclusions:** In both groups, the time to CCyR was longer and long-term results were inferior in comparison with standard patients but the differences were not significant and in accordance to published data. The 12 months follow-up after the study’s end showed that 26 patients were alive and in long-term CCyR and 2 deaths were reported.

**Abbreviations:** CML = Chronic Myeloid Leukemia, BCR-ABL1 = Break Cluster Region - Abelson gene, TKI = tyrosine kinase inhibitor treatment, ACAs = additional cytogenetic abnormalities, CCyR = complete cytogenetic response, PCyR = partial cytogenetic response, mCyR = minor cytogenetic response, MMR = major molecular response, HSCT = hematopoietic stem cell transplant, HLA = human leukocyte antigens, CP = chronic phase, AP = accelerated phase, BP = blast phase, OS = overall survival, CBA = chromosome banding analysis, +8 = trisomy 8, i(17q) = isochromosome (17q), +Ph = second Philadelphia chromosome, -7 = monosomy 7, -17 = monosomy 17, +17 = trisomy 17, -21 = monosomy 21, +21 = trisomy 21, -Y = loss of Y chromosome, ELN = European LeukemiaNet, IMA600 = Imatinib 600 mg daily, IMA400 = Imatinib 400 mg daily, NILO600 = Nilotinib 600 mg daily, DASA100 = Dasatinib 100mg daily, DASA140 = Dasatinib 140 mg daily

## Introduction

Chronic myeloid leukemia (CML) is a stem cell clonal disease characterized by the acquisition of a fusion protein, *BCR-ABL1* oncogene, which leads to uncontrolled proliferation of myeloid elements in all stages of differentiation [**1**,**2**]. The fusion gene is the result of reciprocal translocation (9;22)(q34;q11) known as Philadelphia (Ph) chromosome, discovered by Nowell and Hungerford in 1960 [**3**-**5**]. CML is a multiphase disease, which may be diagnosed in three distinct phases: chronic phase (CP), accelerated phase (AP) and blast phase known as blast crisis (BP). Most patients are diagnosed in CP [**6**]. 

CML is the first neoplasm in which the knowledge of molecular lesion has enabled the discovery of targeted therapies. Imatinib mesylate, the first generation of *BCR-ABL1* tyrosine kinase inhibitor (TKI), optimized disease treatment and increased overall survival (OS) from fatal within 3-5 years since diagnosis to a life expectancy of 20 years. During theTKI era, protocols for assessing response and resistance to treatment were developed. Once TKI were established as standard treatment for CML, eight years phase III IRIS trial demonstrated that Imatinib induced a complete cytogenetic response (CCyR) in 87% of the patients with an estimated long-term overall survival (OS) of 85% [**7**]. The latest results reported that 17% of the patients did not achieve a cytogenetic response (primary resistance) while 15% of the patients lost the obtained response (secondary resistance). Overall, Imatinib did not obtain optimal results in 40% of the patients [**7**]. During the Imatinib treatment, the monitoring of clonal evolution proved to be very important. The long term monitoring results indicated that most events such as resistance and/ or progression occurred in the first three years, highlighting the importance of regular monitoring in this phase of treatment [**7**]. One important conclusion of IRIS trial was that the achievement of CCyR within the first 12 months of treatment is associated with long-term optimal response [**7**]. Currently, the only feature correlated with the treatment failure is the presence of additional chromosomal abnormalities (ACAs) which were observed in 5 to 10% CML patients diagnosed in CP. 

## Materials and methods 

The study was conducted in accordance with the applicable regulatory requirements. The protocol was approved by the institutional review board and ethics committee of the participating centre. All the patients gave a written informed consent before participation.

The observational retrospective study collected data from the Hematology and Bone Marrow Transplantation Department of Fundeni Clinical Institute, Bucharest. During 2005 until 2013, 199 CML patients were diagnosed and monitored. In this single centre study, 28 (14.07%) CML patients with ACAs at diagnosis (group A) and during TKI (group B) were included. The aim of this study was to analyze the impact of ACAs on time to achieve CCyR, treatment and overall survival.

CML was confirmed by fluorescence in situ hybridization (FISH) study for BCR-ABL1 of blood interphase cell nuclei followed by chromosome banding analysis (CBA).

Fluorescence in situ hybridization (FISH) study for BCR (22q11.23)/ ABL (9q34) used Dual Color ON BCR/ ABL translocation probe, BCR was marked with green (G) and ABL with red (R). Expected signals patterns: negative 2R 2G (normal) and positive (standard): 1R 1G 2F (presence of BCR/ ABL translocation). The exam analyzed blood interphase nuclei and not metaphases.

Fresh bone marrow samples were obtained. Samples were prepared by using overnight and cell cycle synchronization cultures and were analyzed by using GTG chromosome banding analysis (CBA) [**14**]. At least 20 metaphases were analyzed. The karyotype was described according to International System for Human Cytogenetic Nomenclature (ISCN) 2009 [**15**]. 

According to the European LeukemiaNet (ELN) recommendations, the cytogenetic response (CyR) was evaluated at month 3 and 6 of the TKI treatment, every 6 months until CCyR, every 12 months since CCyR and at any time on a clinical suspicion of progression [**21**].

## Results

During 2005-2013, 199 CML patients were diagnosed and monitored in our centre. Only 28 (14.07%) CML patients with ACAs at diagnosis and during TKIs were reported. The median age of patients was 50.5 years. EUTOS recommendations at the patient’s enrolment were first generation TKI (Imatinib) with a different dose according to phase and risk or inclusion in clinical trials.

In the study, we evaluated 11 (5.52%) patients with ACAs at diagnosis (group A) and 17 (8.54%) patients with ACAs during the TKI treatment (group B). 

**Table 1:** Characteristics of patients in group A and B

Parameters		Group A (N=11)	Group B (N=17)
Males		7	8
Females		4	9
Median age (years)		51.72 (32-75)	49.70 (25-71)
CML-CP		9	17
CML-AP		2	0
EUTOS score	low	9 (81.81 %)	16 (94.11 %)
	high	2 (18.18 %)	1 (5.88 %)
Cytogenetic abnormalities in Ph positive cells	minor route	8 (4.01 %)	4 (2 %)
	major route	3 (1.50 %)	5 (2.51 %)
Cytogenetic abnormalities in Ph negative cells	minor route	0 (0 %)	5 (2.51 %)
	major route	0 (0 %)	3 (1.50 %)

In group A, 11 (5.52%) patients were included. Nine patients were in CP and two patients in AP. Retrospective EUTOS score were low in all CP patients. Nine patients received Imatinib and two patients Nilotinib. During follow-up, one patient was in lymphoid BP, two patients in AP, and eight patients in CP according to ELN recommendations.

The patient in lymphoid BP received induction therapy but died due to disease progression, two patients in AP received Dasatinib and eight patients in CP received as it follows: two patients maintained Nilotinib and six patients received Imatinib. The median time to CCyR was of 20 months. At the study’s end according to ELN recommendations, 9 patients were in CCyR, 1 patient received allogeneic hematopoietic stem cell transplant (HSCT) from unrelated HLA matched donor and 1 death was reported. 

**Table 2:** Characteristics of patients in group A

Pts no.	CML phase at diagnosis	CML phase at progression	TKI at diagnosis	TKI after progression	TKI at study`s end	Cytogenetic response at study`s end	Survival at study`s end
1	AP	CP	IMA600	IMA400	IMA400	CCyR	yes
2	CP	AP	IMA400	DASA 140	DASA 140	CCyR	yes
3	CP	CP	IMA400	IMA400	IMA400	CCyR	yes
4	CP	CP	NILO600	NILO600	NILO600	CCyR	yes
5	CP	AP	IMA400	DASA 140	allogeneic HSCT	CCyR	yes
6	CP	lymphoid BP	IMA400	N/A	induction therapy	N/A	no
7	CP	CP	IMA400	IMA400	IMA400	CCyR	yes
8	CP	CP	IMA600	IMA400	IMA400	CCyR	yes
9	AP	CP	IMA600	IMA400	IMA400	CCyR	yes
10	CP	CP	NILO600	NILO600	NILO600	CCyR	yes
11	CP	CP	IMA400	IMA400	IMA400	CCyR	yes

Of eleven patients, eight expressed minor route ACAs and three expressed major route ACAs. The most frequent minor route cytogenetic abnormalities in Ph positive cells were monosomies and structural abnormalities (inversions and translocations). The most frequent major route cytogenetic abnormalities in Ph positive cells were trisomy 8 (27.27%), trisomy 19 (9.09%) and second Philadelphia chromosome (9.09%). No minor route abnormalities of chromosome 7 in Ph negative cells were identified.

**Table 3:** Karyotype of patients in group A according to 2009 ISCN recommendations

Pts no.	Karyotype at diagnosis
1	46,XY,t(9;22)(q33;q11)[13]/46,XY,t(9;22)(q34;q11),-15,+19[5]/ 52,XY,t(9;22)(q34;q11),+8,+12,+13,+14,+19,+20[4]
2	46,XX,t(9;22)(q34;q11)[12]/45,X,-X,t(9;22)(q34;q11)[4]/46,XX[6]
3	46,XY[16]/46,XY,t(9;22)(q34;q11)[8]/44,XY,t(9;22)(q34;q11),-15,-19[3];
4	46,XY,t(9;22)(q34;q11)[2]/46,XY,t(9;22)(q34;q11),inv(1)(p?;q?)[13]/46,XY[5];
5	46,XY,t(9;22)(q34;q11)[15]/46,XY,t(1;9;22)(q?;q34;q11)[7];
6	46, XX,t(9;22)(q34;q11)[8]/45,X,-X,t(9;22)(q34;q11)[4]/46,XX[9];
7	46,XX,t(9;22)(q34;q11),t(2;7)(q21;q22)[18]/ 46,XX[4]
8	46,XY,t(9;22)(q34;q11),t(12;14)(q22;q24)[8]/47,XY,t(9;22)(q34;q11),t(12;14) (q22;q24),+8[2]/48,XY,t(9;22)(q34;q11),t(12;14)(q22;q24),+8,+der(22)t(9;22)[10]
9	46,XX,t(9;22)(q34;q11)[19]/97,idemx2,+der(22)(q34;q11)[3]
10	46,XY,t(9;22)(q34;q11)[21]/45,X,-Y,t(9;22)(q34;q11)[4]/46,XY[9]
11	46,XY,t(9;22)(q34;q11)[19]/45,X,-Y,t(9;22)(q34;q11)[3]/46,XY[5]

In group B, 17 (8.54%) patients were included. All patients were in CP of CML at diagnosis. The retrospective EUTOS score were low for 16 patients and high for 1 patient. All patients received Imatinib. The median duration of the TKI treatment until ACAs detection was of 36 months. 

At the ACAs detection, 5 patients received Dasatinib and 11 patients Imatinib. The patients maintained Imatinib 400 mg daily due to intolerance to Dasatinib and higher dose Imatinib. One patient received a higher dose of Imatinib and Hydroxicarbamide due to intolerance to Dasatinib. For this patient, no related and unrelated HLA matched donors were available. At the time, Nilotinib was available as first line in clinical trials but not in second line.

During follow-up, one patient had progressed to myeloid BP, six patients in AP, and ten patients in CP according to ELN recommendations.

The patient in myeloid BP received induction therapy but died due to neutropenia complications, six patients received allogeneic hematopoietic stem cell transplant (HSCT) from unrelated HLA matched donor and ten patients maintained previous TKI treatment and achieved CCyR in a median time of 24 months.

At the study’s end, according to ELN recommendations, ten patients were in CCyR, six patients received allogeneic hematopoietic stem cell transplant from unrelated HLA matched donors and one death was reported. 

**Table 4:** Characteristics of patients in group B

Pts no.	Initial CML phase	CML phase at progression	Initial TKI	TKI at progression	Cytogenetic response at ACAs detection	Treatment at study end	Survival at study’s end
1	CP	myeloid BP	IMA 400	N/A	failure	induction	no
2	CP	AP	IMA400	DASA140	CCyR	DASA140	yes
3	CP	AP	IMA400	DASA100	mCyR	allogeneic HSCT	yes
4	CP	AP	IMA 400	IMA 600 + Hydroxicarbamide	mCyR	allogeneic HSCT	yes
5	CP	CP	IMA 400	IMA 600	CCyR	IMA 600	yes
6	CP	CP	IMA 400	IMA 400	CCyR	IMA 400	yes
7	CP	CP	IMA 400	IMA 400	CCyR	IMA 400	yes
8	CP	CP	IMA 400	IMA 400	CCyR	IMA 400	yes
9	CP	AP	IMA 400	DASA100	mCyR	allogeneic HSCT	yes
10	CP	AP	IMA 400	DASA100	mCyR	allogeneic HSCT	yes
11	CP	CP	IMA400	IMA400	CCyR	IMA 400	yes
12	CP	CP	IMA400	IMA600	CCyR	IMA600	yes
13	CP	CP	IMA400	IMA400	CCyR	IMA400	yes
14	CP	CP	IMA 400	IMA400	CCyR	IMA 400	yes
15	CP	AP	IMA 400	DASA100	PCyR	allogeneic HSC	yes
16	CP	AP	IMA 400	DASA100	PCyR	allogeneic HSC	yes
17	CP	CP	IMA 400	IMA400	CCyR	IMA 400	yes

Of 17 patients, 5 patients expressed major route ACAs, 3 patients expressed major route cytogenetic abnormalities in Ph negative cells, 4 patients expressed minor route ACAs and 5 patients expressed minor route cytogenetic abnormalities in Ph negative cells. The most frequent major route cytogenetic abnormalities in Ph positive and negative cells were trisomy 8, trisomy 19 and second Ph chromosome. The most frequent minor route cytogenetic abnormalities in Ph positive and negative cells were marker chromosomes and structural abnormalities (inversions, translocations, and dicentric chromosomes). No minor route abnormalities of chromosome 7 in Ph negative cells were identified.

**Table 5:** Karyotype of patients in group B according to ISCN 2009 recommendations

Pt	TKI until ACAs (months)	Karyotype at ACAs detection
1	60	47,XX,+der(9),idic(22)t(9;22)(q43;q11)[11]/48,XX,+der(9),2idic(22)t(9;22)(q43;q11) [2]/47,XX,+19[3]/46,XX[4];
2	24	46,XY[21]/46,XY,t(9;22)(q34;q11),inv(2)(p?;q?)[2]
3	12	46,XY[15]/46,XY,t(9;22)(q34;q11)[4]/47,XY,t(9;22)(q34;q11),+19[3]
4	60	46,XY,t(9;22)(q34;q11)[16]/44,XY,-21,-22[5]
5	60	46,XX[22]/46,XX,t(9;22)(q34;q11)[1]/45,XX,idem,-18[3]
6	48	46,XY[23]/47,XY,+mar[3]
7	60	46,XY[17]/47,XY,t(9;22)(q34;q11)+8[3]
8	18	46,XX[19];45,X,-X[5]
9	24	46,XY,t(9;22)(q34;q11)[4]/47,XY,t(9;22)(q34;q11),+der(22)t(9;22)[12]/46,XY[4]
10	84	46,XX,t(9;22)(q34;q11)[26]/47,XX,t(9;22)(q34;q11),+mar,+dm[2]/ 46,XX[6]
11	24	46,XX[11]/49,XX,+8,+19,+19[4]/ 49,XX,+8,+14,+19[5]
12	24	46,XY[22]/47,XY,+8[3]
13	24	46,XY[18];47,XY,+mar[2]
14	12	46,XX,t(9;22)(q34;q11)[12]/46,XX,t(9;22)(q34;q11),+8,-13[4]/45,XX,dic (3;17)(q?;p?)[1]/46,XX[8];
15	12	46,XY[19]/47,XY,t(9;22)(q34;q11),+mar[4]
16	6	46,XX[16];46XX,t(9;22)(q34;q11)[10];47,XX,t(9;22)(q34;q11),+19[4]
17	60	46,XX[19]/ 45,XX,-21[4]

## Discussions

The 2013 ELN recommendations suggest that the presence of ACAs at diagnosis is a warning signal. ACAs during the TKI treatment define TKI failure and are associated with shorter overall survival (OS) on second-line Imatinib [after Interferon alpha (rIFNα) first line treatment] but not second-line Dasatinib or Nilotinib. ACAs have been reported to have an adverse prognostic value, particularly in the case of major route abnormalities including trisomy 8, trisomy Ph (+der(22)t(9;22)(q34;q11)), isochromosome 17 (i(17)(q10)), trisomy 19 and ider(22)(q10)t(9;22)(q34;q11). High-risk and major route ACAs can help identify patients eligible for investigational approaches but in daily practice, they do not mandate different initial treatments. Major route ACAs developing during treatment were confirmed to be a signal of acceleration. Cytogenetic abnormalities in Ph negative cells occur in 5% to 10% of the patients and, in the absence of dysplasia, do not seem to adversely affect the outcome. The exception is abnormalities of chromosome 7 (monosomy 7 and del (7q)). Some case reports indicate a risk of myelodysplasia and acute leukemia and justify long-term follow-up bone marrow biopsies [**21**].

Two large studies have confirmed the prognostic impact of ACAs detection. An Italian Working Group study which evaluated 378 patients, detected the presence of ACAs in 21 (5.6%) patients for whom the time to achieve CCyR and major molecular response (MMR) were longer. Long-term results in patients with ACAs were inferior but the differences were not significant compared to standard CML patients [**8**]. A German working group study identified the presence of ACAs in 79 (6.9%) of 1151 enrolled and Imatinib treated patients. For those patients, the time to achieve CCyR and MMR was longer. Progression free (PFS) and overall survival (OS) were shorter than in patients with standard t (9; 22) [**9**]. 

The presence of ACAs at diagnosis and during TKI treatment may announce treatment failure and/ or transformation to advanced stage (accelerated or blast) [**10**]. ACAs can be divided into major route ACAs represented by trisomy 8 (+8), isochromosome (17q)[i(17q)], trisomy 19 (+19) and a second Philadelphia chromosome (+Ph) and minor route ACA represented by monosomy 7 (-7), monosomy 17 (-17), trisomy 21 (+21), loss of Y chromosome (-Y) and t (3; 21) (q26; q22) [**11**,**12**]. At least one of those abnormalities occurs in 15% of Philadelphia positive cells. Multiple cytogenetic abnormalities (numerical and structural) were associated with translocation (9;22). The most common chromosomal abnormalities are represented by trisomy 8 (+8) (34%), second Philadelphia chromosome (+ Ph) (30%), isochromosome (17q) (17q) (20%), trisomy 19 (+19) (13%), loss of Y chromosome (-Y) (8%), trisomy 21 (+21) (7%), monosomy 17 (-17) (5%) and monosomy 7 (-7) (5%) [**13**]. Clonal evolution in CML is a marker of progression and represents chromosomal instability [**16**]. Shortening of telomere length is frequently associated with disease progression and may be a factor involved in chromosomal instability as telomere length is an important factor in chromosome integrity [**16**]. 

**Table 6:** Patterns of additional chromosomal abnormalities in CML

Chronic phase (according to frequency)	Accelerated and blastic phase (according to frequency)
classic +Ph	classic +Ph
variant +Ph	variant +Ph
+Ph, +8	+Ph, +8
+Ph, -Y	+Ph, -Y
+Ph, -21	+Ph, i(17q)
+8	+19/ +17/ -7
complex karyotype	complex karyotype

From 11 (5.52%) patients in group A, eight expressed minor route ACAs and three expressed major route ACAs. The most frequent minor route ACAs were monosomies and structural abnormalities (inversions and translocations). The most frequent major route ACAs cells were trisomy 8 (27.27%), trisomy 19 (9.09%) and second Philadelphia chromosome (9.09%). Our results are similar to those published in the literature [**9**,**17**]. Major route ACAs discovered at diagnosis may alert to early progression to advanced phase (accelerated or blast) and are important for assessing time to CCyR. Minor route ACAs have not yet proved important for progression [**18**]. 

From 17 (8.54%) patients in group B, 5 patients expressed major route ACAs, 3 patients expressed major route cytogenetic abnormalities in Ph negative cells, 4 patients expressed minor route ACAs and 5 patients expressed minor route cytogenetic abnormalities in Ph negative cells. The most frequent major route cytogenetic abnormalities in Ph positive and negative cells were trisomy 8 (17.64%), trisomy 19 (11.76%) and second Ph chromosome (5.88%). The most frequent minor route cytogenetic abnormalities in Ph positive and negative cells were marker chromosomes and structural abnormalities (inversions, translocations, and dicentric chromosomes). The impact of cytogenetic abnormalities in Ph negative cells is still to be determined but they have been described in 2-17% of the patients on Imatinib [**19**]. This phenomenon is transient and the clinical impact is unclear [**20**].

In both groups, no minor route abnormalities of chromosome 7 in Ph negative cells were reported.

In our study, the median time to BP progression was of 40 months (8-72).

At the end of our study, from 28 patients enrolled, 19 patients were in long-term CCyR on TKI treatment, 7 patients received allogeneic hematopoietic stem cell transplant from unrelated donor and 2 deaths were reported. The 12 months follow up after the study’s end showed that 26 patients are alive and in long-term CCyR. The time to CCyR was longer in both groups and long-term results in patients with ACAs were inferior compared to standard patients but the differences were not significant and in accordance to published. 

## Conclusions

The presence of ACAs at diagnosis and during the TKI treatment may announce treatment failure and/ or transformation to advanced stage (accelerated or blast).

In our study, the cytogenetic response is heterogeneous. The time to CCyR was longer in both groups and long-term results in patients with ACAs were inferior, but the differences were not significant and in accordance to published data. The follow up after the study’s end showed that 26 patients are alive and in long-term CCyR: 19 patients were in long-term CCyR on TKI treatment, 7 patients received allogeneic hematopoietic stem cell transplant from unrelated donor and 2 deaths were reported.

The detection of ACAs during the TKI treatment is a warning sign of disease progression and periodic cytogenetic monitoring is mandatory, allowing real time therapeutic intervention (second generation of TKI or allogeneic hematopoietic stem cell transplant).

In this study, we demonstrated the importance of cytogenetic monitoring for the detection of disease progression and early TKI treatment switch or allogeneic stem cell transplantation. 

**Acknowledgment**

This work was supported by the grant PN 41-087/2007 from the Romanian Ministry of Research and Technology. The authors express the gratitude to European LeukemiaNet for their permanent support.

**Disclosures**

None
